# Dental Irritation

**Published:** 1852-01

**Authors:** H. E. Peebles


### DENTAL IRRITATION.

*A case of excessive pain, occasioned by the introduction of foreign substance between the teeth.*

XENIA, Nov. 10th, 1851.

About the 20th of August I was called upon to examine the teeth of a lady, who stated that she had been suffering for several days with tooth-ache, and very severely for the last three days. She supposed the pain originated in the second superior molar of the right side. Upon examination it proved to be but slightly decayed, and that on the posterior approximal surface, penetrating but little beneath the enamel. The posterior surface of the first molar was very much decayed. The entrance to this decay was quite small, a round orifice at the posterior edge of the crown of the tooth. By introducing an instrument and examining all about the walls of the cavity, no sensative point could be found, and percussion upon the teeth with a steel instrument produced no marked effect. The character of the pain was not that experienced from an inflamed nerve. It appeared to originate in the outset with these teeth, or the second molar, as she remarked; but the pain, soon after its first attack, extended over that entire side of the face and head, including the eye of that side, the ear, and the lower jaw almost to the symphysis, involving the submaxillary gland, and extending a considerable distance down the neck. The pain she represented as being most excruciating. It was evident, upon examination, that it did not proceed from inflammation of the nerve, occasioned by decay, nor from inflammation of the external periosteum, nor from disease of the antrum. There being no indication of these things, I was at a stand to know what course to pursue. I could perceive no indication for extraction. I separated the first and second molars by a large space, with a view to expose the decay more perfectly, when taking an excavator I found the cavity as before. Upon introducing the instrument down farther between the necks of the teeth, I detected and removed some hard substance, which proved to be the seeds of the blackberry, three of which I

removed from that place. There was also along with them a piece of the enamel of the crown of the tooth, equal in size to the seeds. The orifice into the cavity was slightly larger than one of the seeds. The seeds had been forced into this orifice, and down into their position, while the lady had been eating blackberries, about ten days previous to their removal. Immediately upon the removal of these, the pain ceased and has not since returned, now about three months. The side of the face and head was very sore after the pain ceased. This gradually subsided, and in a short time was entirely gone. After separating the teeth and ascertaining the cause of the difficulty, I cleansed and filled the cavity in the first molar.

This case presents some rather peculiar features: first, that these substances should have remained after their introduction some five or six days without causing any uneasiness. Their introduction into the decayed cavity of the tooth can easily be understood, but how they should all have been compressed so far down between the teeth, I confess I cannot so easily perceive; and more peculiar is it still, that the difficulty should have been so great, the pain so intense, and yet no apparent inflammation of the periosteum, gums, or any of the contiguous parts. Are cases of this kind rare? I have met with but one before of a similar character. It was caused by the introduction of several pieces of broken tooth, down between the necks of the teeth. Things of this kind admonish us to make our examinations very thorough, and especially when we have any doubts as to the cause of any difficulty; and also perfectly to acquaint ourselves, so far as possible, with the distinctive indications of the various diseases to which the teeth and their approximate parts are subject. J. Taft.

### TO THE YOUNG DENTIST IN THE WEST.

My Young Brother—Having lately embarked in the dental art, you thereby become affilliated with, not only the writer, but the entire profession. You are now enrolled for a voyage of usefulness ; your name is inscribed upon our banner, your fortune "for weal or for woe" is cast with our's. I know not what your opportunities have been, or what are your qualifications; I know not what is your aim or where lies your goal, but this I know, that you have entered the ranks of an honorable, an useful, and responsible avocation, and now may claim some of the noblest spirits, the brightest intellects, the most devoted, self-denying, indefatigable men of our country as your brothers; but remember that they do not give glory and luster to you, unless you present a brightness and polish to the beams of light radiating from them, that may reflect the same off, to the eyes of community. For if you do not possess the polish of a gentleman, and brightness of a good man, or the luster of merit, but the dark, rough, homely deformity of a selfish, narrow-minded, ignorant, soulless quack, or puffing charlatan, you absorb the light and glory of your elder and better brothers, and thereby rob the world of every ray that strikes your sphere. Yon may have embarked hastily; you may have not duly considered the consequences; you may not be duly and truly prepared, worthy and well qualified, for the voyage of professional life; you may not have started aright; you may have failed to count the cost; you may not have looked to the end from the beginning; perhaps you merely blundered into our ranks; or you may have, at a late period, quit a good and useful trade, for which you were by nature and education competent, merely because our art offered, as *you* supposed, a better reward for your labor, I know not: but you know how the case stands with you, and if you feel and know you are disqualified by nature, taste, or inclination, stop, and for the love of truth, honesty, and your fellow-men, abandon our ranks, quit our society at once and *forever*, for my word for it, you can *never* succeed. You are guilty of robbery of a two-fold character—

you rob your patron of his money and teeth, and the profession of its honors and patronage ; you are a mere leech, a stain, a scab.

If you have been deceived by your preceptor, (as is the case sometimes,) believing him to be one of the best, and he turned you out with the assurance that you were well qualified, (and some men of reputation have done so, and even given certificates of qualification to students of a few months, when they must have known they were in error,) which you now find not true; or if you commence practice in the belief that little skill and less preparation were necessary, and find you were grossly mistaken ; or if you see jobs done better than you can do them, and have a laudable ambition to excel, an abiding love for the art, a desire to do good and be useful to the human family and an honor to the profession, the nerve to proceed along the thorny path of attainment, and the determination to succeed notwithstanding obstacles oppose ; press on, upward and onward; go to the source of dental light, the fountain of dental knowledge; enter one of our excellent schools, and then drink deeply in the stream of knowledge; receive and reflect the light of experience, and learn to compete successfully with the best, the oldest, and receive authority from the hands of a delighted faculty, to go abroad in the world and carve out for yourself a character among your peers, and build up a reputation among the people.

But should you have done all that you could to start aright, having had good precepts, well read, schooled and authorized, and are you a good man and true, and does not practice increase, cases come ; does not the merited reward of honest labor flow into your needy hand; are you pressed for means to adorn and beautify your rooms ; or do you even find it a hard matter to procure the necessary material, instruments and fixtures, be not discouraged, cast down, or disheartened ; persevere, attend to your office closely, do *all* your work well and faithfully, and in a manner to satisfy *yourself*. Turn not off a job done "well enough," but as well as *you can do it*, and strive to do the present better than the last, and remember the man to make a dentist should be a man of sense, of sound judgment, a good mechanic, honest, firm, decided, truthful, plain, candid, studious, intelligent, discreet, prudent, moral, temperate, neat, modest, polite, affable, persevering, aspiring, in a word, a gentleman.

Having these qualifications, select a location where wealth and intelligence are, no matter how much competition you have, and you are bound to SUCCEED. H. E. PEEBLES.

*Lexington, Mo.*

